# Supplementary material for: Heparin Protects Severe Acute Pancreatitis by Inhibiting HMGB-1 Active Secretion from Macrophages
Source: Polymers (Basel). 2022 Jun 17;14(12):2470. doi: 10.3390/polym14122470 (PMC9227308; doi:10.3390/polym14122470)
Supplement: Supplementary file 1 [file polymers-14-02470-s001.zip › polymers-1739286-supplementary.pdf]

Electronic Supplementary Material for

Heparin Protects the Severe Acute Pancreatitis by Inhibiting HMGB-1 Active Secretion from Macrophage

Jing Yang [1], Xujiao Tang [1], Qingqing Wu [1], Panpan Ren [1], Yishu Yan [1,*], Wei Liu [2] and Chun Pan [3]

[1] School of Life Sciences and Health Engineering, Jiangnan University, Wuxi 214122, China; yangjing@jiangnan.edu.cn (J.Y.); 6201507011@stu.jiangnan.edu.cn (X.T.); 6191502015@stu.jiangnan.edu.cn (Q.W.); 6201507007@stu.jiangnan.edu.cn (P.R.)

[2] Jiangsu Key Laboratory of Druggability of Biopharmaceuticals, State Key Laboratory of Natural Medicines, School of Life Science and Technology, China Pharmaceutical University, Nanjing 210009, China; liuwei@cpu.edu.cn

[3] Department of Critical Care Medicine, Zhongda Hospital, Southeast University, Nanjing 210009, China; panchun1982@gmail.com

* Correspondence: yanyishu@jiangnan.edu.cn

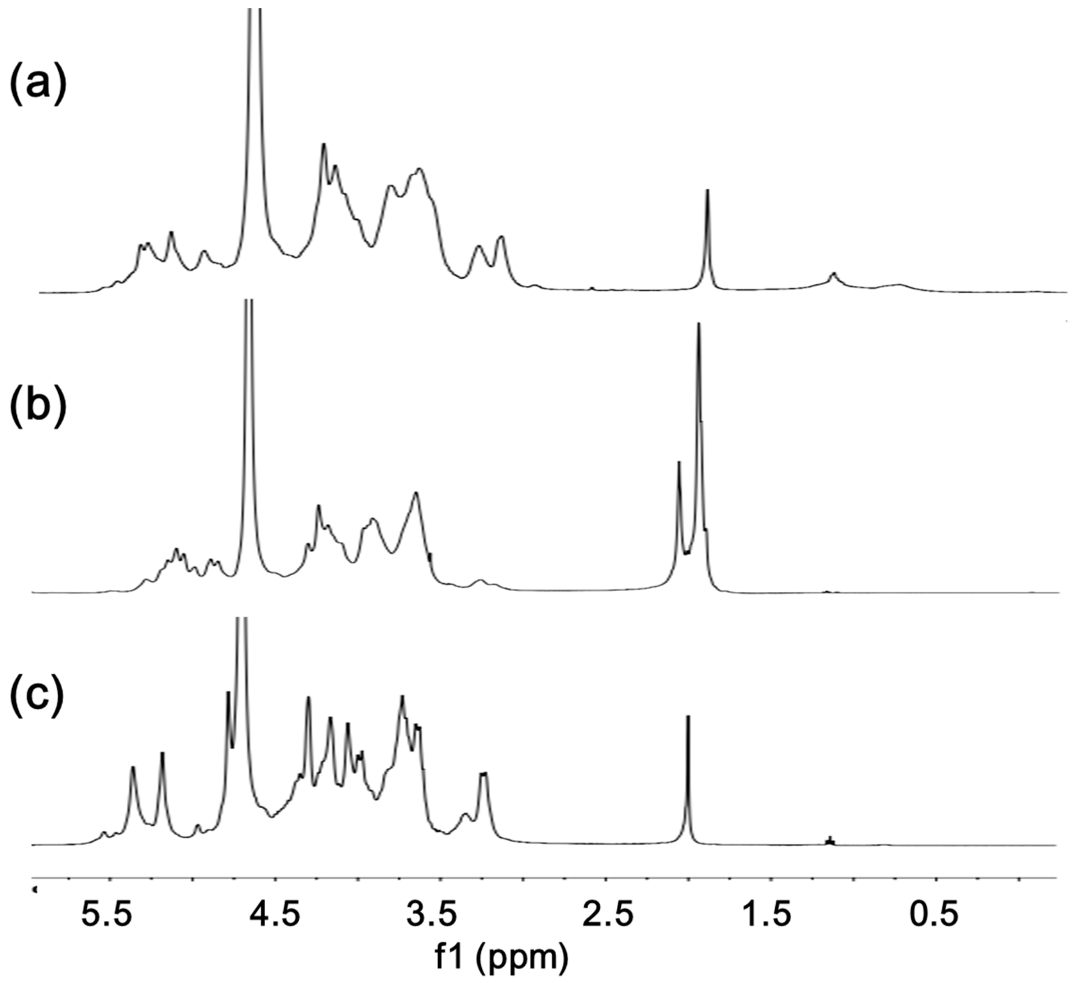

Figure S1. Comparison of ^{1}H NMR spectrum of heparin and 6-desO H and N-Ac H.

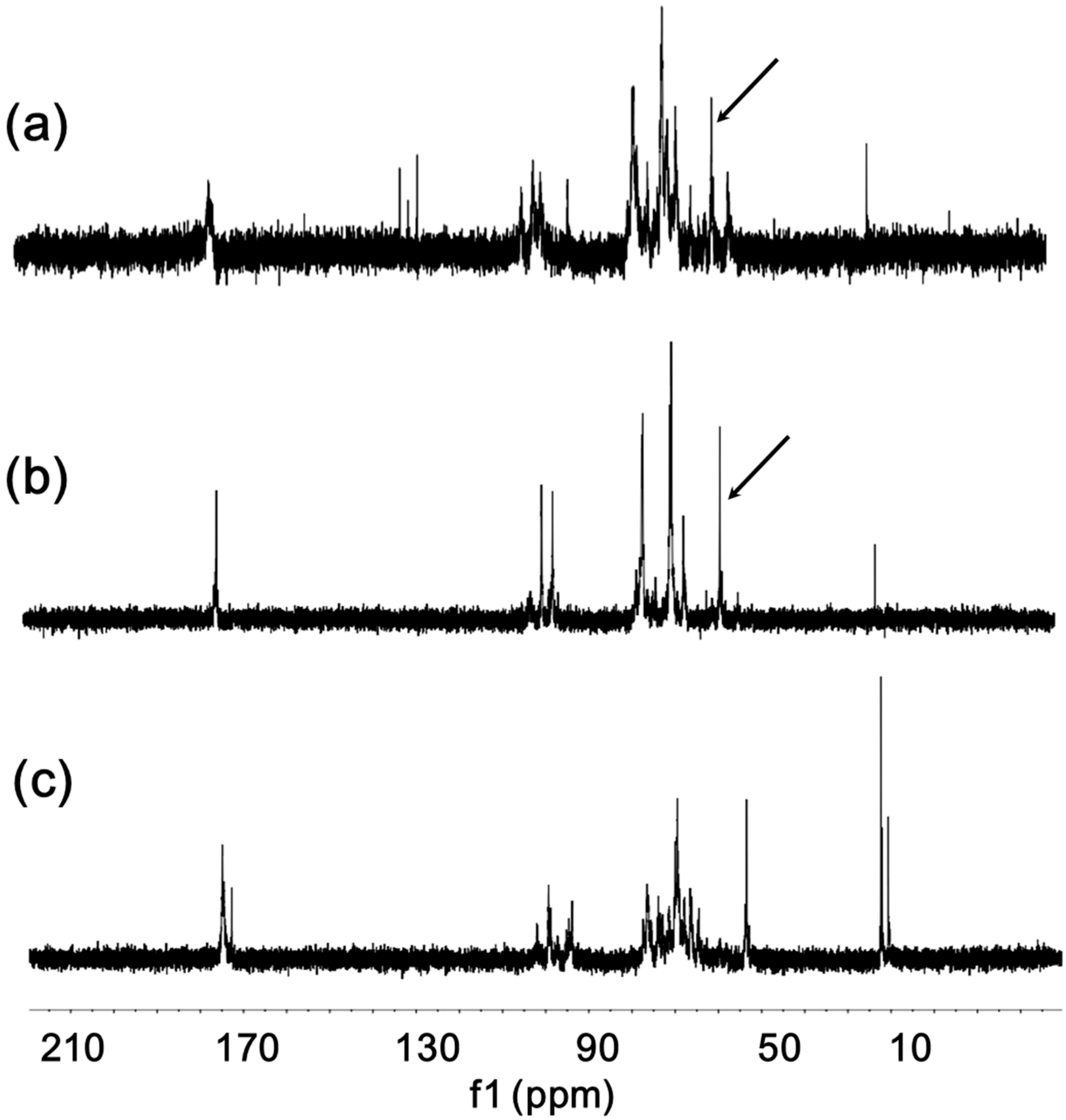

Figure S2. Comparison of ^{13}C NMR spectrum of heparin and 6-desO H and N-Ac H.

Table S1. Molecular weight distributions of the heparin derivatives

**Sample**	**Mw**	**Mn**	**Mw/Mn**	**Mw distribution(%)**			
				＞24k	16k-24k	8k-16k	＜8k
**Heparin**	17375	14994	1.155	16.64	36.09	42.01	5.26
**6-desOH**	15271	13170	1.16	10.24	26.03	53.11	10.62
**N-acetyl H**	15722	13750	1.143	9.75	31.14	51.42	7.69
